# Role of physical activity in cardiovascular disease prevention: impact of epigenetic modifications

**DOI:** 10.3389/fcvm.2025.1511222

**Published:** 2025-01-20

**Authors:** Yi Sun, Zuoying Peng, Hua Liang

**Affiliations:** ^1^ Heilongjiang Academy of Traditional Chinese Medicine, Harbin, China; ^2^ School of Basic Medicine, Heilongjiang University of Chinese Medicine, Harbin, China

**Keywords:** cardiovascular disease, physical activity, epigenetic, non-coding RNA, sedentary lifestyle

## Abstract

Cardiovascular disease (CVD) remains the leading cause of death worldwide, imposing a major burden on morbidity, quality of life, and societal costs, making prevention of CVD a top public health priority. Extensive research has pointed out that lack of adequate physical activity in life is one of the key risk factors for heart disease. Indeed, moderate exercise is not only beneficial to the heart in healthy populations, but also exerts a protective effect in pathological states. However, the molecular mechanisms underlying the cardioprotective effects of exercise are still not fully understood. An increasing body of research indicates that variations in the epigenetic system—such as DNA methylation, histone modifications, and production of non-coding RNA—are essential for maintaining heart health and preventing heart disease. Exercise is a potent epigenetic modulator that induces direct and long-lasting genetic changes and activates biological signals associated with cardiovascular health. These changes can be influenced by external stimuli such as physical activity and may even be passed on to offspring, thus providing a mechanism for generating genetic effects through behavioral interventions. Therefore, understanding this relationship can help identify potential biomarkers and therapeutic targets associated with CVD. This study aims to provide an overview of the beneficial effects of exercise on heart health. This information may help guide future research efforts and improve our understanding of epigenetics as a therapeutic, prognostic, and diagnostic biomarker for CVD.

## 1 Introduction

With almost 18.6 million deaths annually from cardiovascular disease (CVD), it is the leading cause of death globally (1). CVD encompasses a range of conditions that affect the heart and blood vessels, including coronary artery disease, heart failure, congenital heart defects and peripheral artery disease (2). Major risk factors for these diseases include physical inactivity, smoking, high alcohol intake and elevated cholesterol levels. With the growing aging of the population worldwide, the incidence and mortality of CVD is increasing annually, with the age of the disease becoming progressively younger, and posing a significant burden on the quality of life and social costs (3).

Epigenetic regulation affects gene activity and expression associated with CVD through DNA methylation, histone modifications, and regulation of non-coding RNAs (ncRNAs) (4). Epigenetic marks serve as key molecular indicators for the early diagnosis of CVD and involve critical pathways (5). Due to the reversible nature of these modifications, the genes and proteins involved provide novel target sites for CVD therapy, stimulating extensive research interest in therapeutic approaches based on epigenetic strategies.

According to the World Health Organization, a sedentary lifestyle coupled with consistently low levels of physical activity increases the risk of CVD and shortens life expectancy, which are several risk factors for the onset and course of CVD (2). Clinical studies, cohort studies, systematic evaluations and meta-analyses have demonstrated the beneficial effects of exercise in reducing cardiovascular risk factors and the risk of cardiovascular events (6, 7). Exercise as an important lifestyle modifier positively affects cardiovascular health by influencing epigenetic mechanisms (8).

With increased research on the epigenetics of CVD, the epigenetic effects of combined exercise interventions have been widely studied. However, to the best of our knowledge, the possible biological mechanisms of how exercise may ameliorate CVD disease through epigenetic inheritance have not been fully examined. Therefore, we summarize the epigenetic landscape associated with physical activity in heart health and illness, information that will provide new perspectives and strategies for CVD prevention and treatment.

## 2 Overview of epigenetic changes

Epigenetic mechanisms are adaptive parts of the genome that modify gene function in response to external factors and ensure continuity of gene expression profiles across cell generations (9). These modifications are usually categorized into three main types: chemical modifications of DNA, such as DNA methylation, which is a common modification that regulates gene activity by adding methyl groups. Alterations in chromatin structure, which include various chemical modifications of histones, such as acetylation and methylation, that alter the compactness of chromatin and thus affect gene expression. Finally, ncRNAs, especially microRNAs (miRNAs), which regulate gene expression by affecting the stability or translational efficiency of mRNAs, thereby modulating gene function without the need to alter the DNA sequence (9, 10).

### 2.1 DNA methylation

DNA methylation is an epigenetic change that refers to the attachment of a methyl group to DNA nucleotides, primarily found in nuclear DNA. In DNA, methylation of cytosine to form 5-methylcytosine (5mC) occurs predominantly at the CpG site, the sequence in which cytosine is followed by thymine. In human cells, approximately 60%–80% of the CpG site is methylated. DNA methylation is commonly associated with gene silencing, as it inhibits gene expression by reducing chromatin accessibility and hindering the function of DNA-binding proteins such as transcription factors. In addition, certain factors such as nutrition or exercise can affect methylation patterns, leading to hypermethylation or hypomethylation of gene promoter regions (11).

CpG islands are CpG-dense regions located near the 5' transcriptional start sites of genes; these regions are typically of low methylation status and are of high regulatory importance for gene expression (11). In contrast, the methylation state of CpG island banks is more variable, and these regions are equally critical for the regulation of gene expression and repression of reverse transcriptional transposons. The primary enzymes responsible for catalyzing DNA methylation are known as DNA methyltransferases (DNMTs), which comprise DNMT1, DNMT3A, and DNMT3B (12). DNMT1 is primarily responsible for maintaining existing methylation patterns, especially during DNA replication, recognizing hemimethylated DNA and maintaining the methylated state. In contrast, DNMT3A and DNMT3B enzymes promote initial methylation of previously unmethylated DNA regions.

In addition to 5mC, methylation modifications of several other intermediate forms, such as 5-hydroxymethylcytosine, 5-formylcytosine, and 5-carboxycytosine, as well as methylation of adenine (6 mA), which is commonly found in bacteria, have been identified and reported in eukaryotes (13). These findings extend our understanding of the diversity and complexity of DNA methylation. Furthermore, DNA methylation is not limited to nuclear DNA but is also present in mitochondrial DNA and RNA, and these methylation events may be critical for certain key aspects of cellular function, such as regulating nuclear-mitochondrial interactions through bidirectional communication of methylation and influencing post-transcriptional regulation of RNA.

### 2.2 Histone modifications

Histone modifications play a crucial role in regulating gene activity in the core structure of DNA. Nucleosomes are composed of histones H2A, H2B, H3 and H4, which modify their interaction with DNA through various post-translational changes such as methylation, acetylation, phosphorylation and ubiquitination. These changes affect chromatin state and thus control gene transcription. Specific modifications (e.g., H3K4me1) tend to activate genes, while others (e.g., H3K9me3) usually result in gene silencing (4).

These histone changes are managed by enzymes that add or remove these chemical marks, known as “writers” and “erasers.” This group of enzymes includes histone acetyltransferases and deacetylases (HDACs), which modulate the level of acetylation and affect the structure and accessibility of chromatin. Ongoing histone modifications contribute to the formation of an “epigenetic memory” that preserves patterns of gene expression during cell division and differentiation (9).

### 2.3 Non-coding RNA expression

In the human genome, the majority of DNA sequences (more than 97%) do not directly encode proteins; however, about 80% of non-coding regions are highly transcriptionally active in certain cell types, and these regions transcriptionally generate ncRNAs including tRNAs and rRNAs, which have diverse structures and functions. Functionally regulated ncRNAs such as miRNAs, small interfering RNAs, Piwi intercalating RNAs, and long-chain noncoding RNAs (lncRNAs) play central roles in biological processes and causally contribute to the development of diseases, such as CVD (11).

miRNAs are widely studied types of ncRNAs, which are transcribed by RNA polymerase II and processed in the nucleus and cytoplasm into mature miRNAs, which usually inhibit protein synthesis by binding to target mRNAs. In addition to miRNAs, lncRNAs and circular RNAs (circRNAs) also have a profound effect on gene expression by interacting with proteins involved in transcriptional regulation (14).

ncRNAs can be categorized into two main groups based on their length: short ncRNAs (sncRNAs, <200 nucleotides) and lncRNAs (0.2 kb to 2 kb). sncRNAs such as miRNAs and piRNAs regulate gene expression mainly by inhibiting the translation of target mRNAs, whereas lncRNAs act by directly interacting with chromatin structures or encoding micropeptides, showing the diversity of regulating gene expression (14, 15). In addition, some lncRNAs generate stable, more tissue-specific and evolutionarily conserved circRNAs through the phenomenon of back splicing.

## 3 Physical inactivity and cardiovascular disease

Sedentary behavior is a substantial modifiable risk factor for CVD and death in general. Extended periods of inactivity contribute to numerous health issues including insulin resistance, vascular dysfunction, a preference for carbohydrate oxidation over fat, a transition from oxidative to glycolytic muscle fibers, reduced cardiorespiratory fitness, and losses in muscle and bone strength (16). This behavior also increases body fat, particularly visceral fat, along with elevated levels of lipids and inflammation markers. Brief reductions in sedentary time can enhance vascular function in the legs, lower blood pressure, and improve glucose and insulin levels after meals. Over time, consistent activity can lead to modest gains in weight management, reductions in waist size, body fat percentage, fasting glucose, HbA1c levels, improved HDL cholesterol levels, and enhanced vascular health.

Premature death and other non-communicable diseases are recognized to be associated with physical inactivity (17). A study analyzed population-attributable risk in 168 countries to estimate how many diseases could be averted if physical inactivity were eliminated. The results showed that the global all-cause mortality and CVD mortality rates due to physical inactivity were 7.2% and 7.6%, respectively (18). In 2016, the physical inactivity rate in high-income countries was 36.8%, significantly higher than in low-income countries at 16.2%. Globally, 6%–10% of premature deaths, type 2 diabetes, coronary heart disease and colon cancer cases are attributed to physical inactivity, and the health-care costs of physical inactivity associated with these non-communicable diseases are estimated at $53.8 billion (19).

A review that included an umbrella of twenty-four systematic evaluations and meta-analyses showed that regular physical activity among seniors (aged 60 and older) correlates with reduced risks of mortality from all causes and cardiovascular events, as well as lower incidence of breast and prostate cancer, fewer fractures, decreased falls, and reduced disability in daily activities (20). This active lifestyle also contributes to slower cognitive decline, lesser occurrences of dementia, including Alzheimer's disease, and reduced depression, leading to improved overall quality of life and cognitive health in older adults. Although regular aerobic and/or resistance training can mitigate some of the adverse physiological effects, a significant amount of sedentary behavior can still have negative physiological effects. Therefore, reducing sedentary behavior is a low-risk strategy.

## 4 Exercise, epigenetics and cardiovascular disease

**Figure 1:**
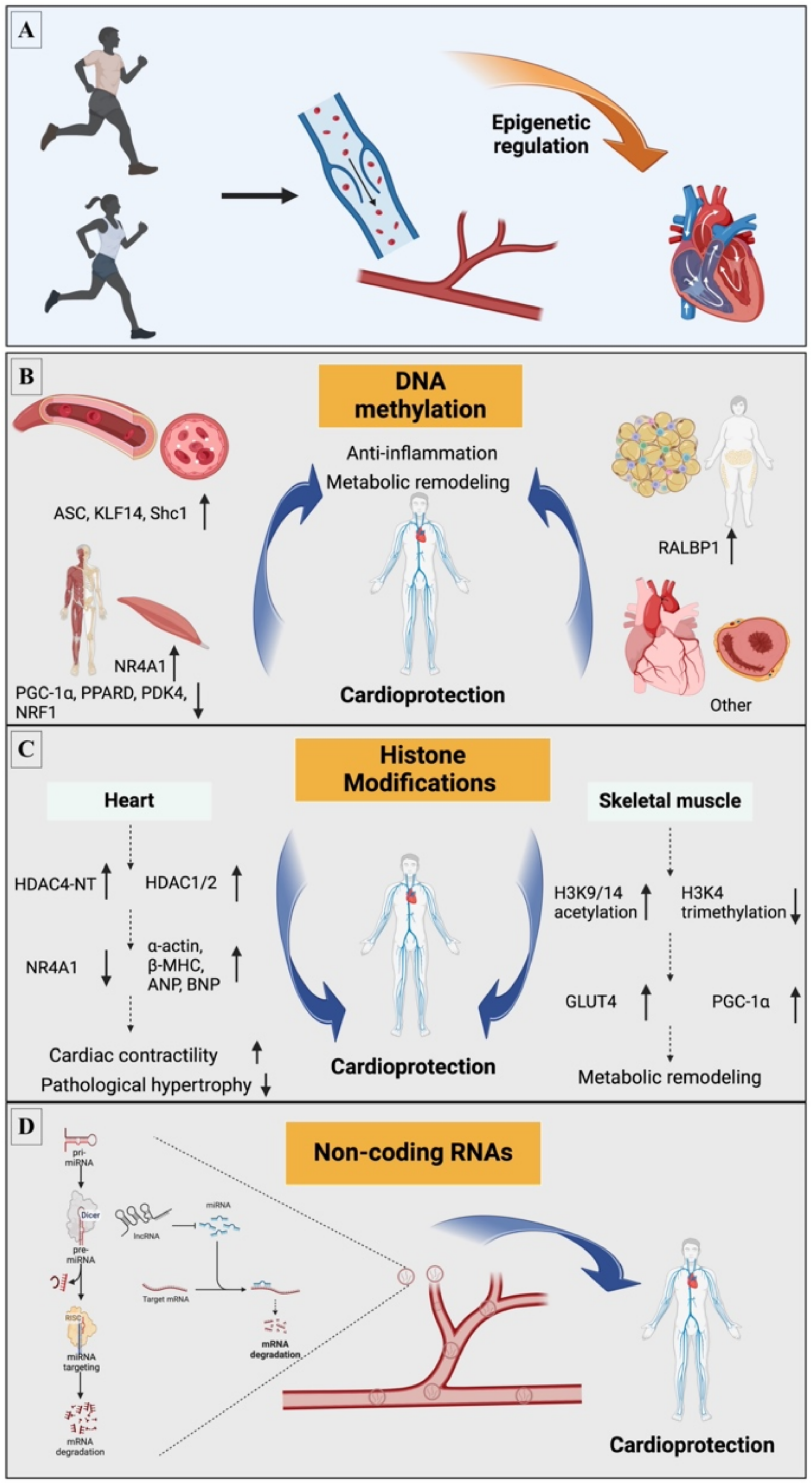
Exercise-induced epigenetic mechanisms involved in CVD. **(A)** Exercise induces CVD through epigenetic mechanisms; mainly involves three aspects: **(B)** DNA methylation; **(C)** Histone modifications; **(D)** Non-coding RNA. ANP, atrial natriuretic peptides; ASC, apoptosis-associated speck-like protein containing a caspase recruitment domain; BNP, brain natriuretic peptides; *β*-MHC, *β*-myosin heavy chain; GLUT4, glucose transporter 4; H3K4, histone H3 lysine 4; H3K9/14, histone H3 lysine 9/14; HDAC, histone deacetylases; HDAC4-NT, HDAC4 N-terminal fragment; KLF14, Kruppel like factor 14; LncRNA, long non-coding RNA; miRNA, microRNA; m, methylation; NRF1, nuclear respiratory factor 1; NR4A1, nuclear receptor subfamily 4 group A member 1; PDK4, pyruvate dehydrogenase kinase 4; PGC-1*α*, peroxisome proliferator activated receptor *γ* coactivator 1 *α*; PPARD, peroxisome proliferator activated receptor *δ*; RALBP1, Ras like proto-oncogene A binding protein 1; Shc1, src homology 2 domain-containing transforming protein C1.

Exercise is a potent epigenetic modulator that triggers immediate and lasting changes that stimulate pathways associated with cardiovascular health (21–26). Specifically, exercise causes a variety of epigenetic modifications, including changes in DNA methylation, post-translational modifications of histones, and ncRNAs, which can directly alter cardiac epigenetic status, improve vascular function, reduce inflammatory responses, alter myocardial metabolism, and protect the heart from aggression (27). Thus, exploring the underlying mechanisms of these protective effects is essential for grasping how exercise contributes to cardiovascular health and slows disease progression (Figure 1, Table 1).

**Table 1:** Epigenetic modifications induced by exercise in cardiovascular disease patients.

Study type	Type of cardiovascular	Disease severity	Number of participants	Study population (Age-years, BMI-kg/m^2^, gender)	Exercise intervention	Exercise session duration	Epigenetic modification	References
CCT	CAD	-	12	66 ± 11; 26 ± 3; 12 males	Exercise (cardiac rehabilitation programme)	60 min (warming up-15, cardiovascular training-30, warming down-15); 2/wk, 10 wk	has-miRNA-92a, has-miRNA-92b △ NDUFA1 and CASP3 ▽	Taurino et al. (2010)
CT	POAD	Fontaine II IC	12	65 ± 9; 25 ± 4; 8 males	Intermittent treadmill walking	Initial: 30 min, progressive increase: + 5 min, every 2 weeks final: 55 min; 3/wk, 12 wk	miRNA-155, miRNA146a, miRNA-200c Unaltered expression miRNA-155, miRNA-200c ▽	Nowak et al. (2012)
RCT	CHF	EF (T): 28 ± 2% EF (UT): 29 ± 1%	Trained (T): 17 Untrained (UT): 17	T: 56 ± 2; 27 ± 1; 13 males UT: 54 ± 2; 27 ± 1; 15 males	CPET	30 min (first 15 days), 40 min (remaining period); exercise intensity: 10% below RCP, 60–72% VO2peak; 3/wk, 4 months.	miRNA-146, miRNA-16 △ miRNA-143 Unaltered expression	Antunes-Correa et al. (2014)
CT	-	-	12 HCS	21.1 ± 2.7; 23.9 ± 2.3; 12 males	Sprint interval training	Week 1: 3 sprints on Day 1, 4 sprints on Day 2, 5 sprints on Day 3 Week 2 onwards: 4 sprints on Day 1, increasing to 8 sprints by the final training session; 3/wk, 4 wk.	mRNA (EGF) △ mRNA (UNG) ▽	Denham et al. (2014)
CT	CHF	EF: 47.68 ± 2.58%	28	59.07 ± 1.79; 26 ± 2; 28 males	CPET	Symptom-limited incremental cardiopulmonary exercise test on a bicycle ergometer	miRNA-21, miRNA-378, miR-940 △	Xu et al. (2016)
CCT	CAD	VO_2_max: 23.6	20	58.0 ± 7.7; 29 ± 5; 10 males	CPET	Symptom-limited incremental cardiopulmonary exercise test on a bicycle ergometer	miRNA-101-3p, miRNA-141-3p, miRNA-200-3p ▽	Mayr et al. (2018)
RCT	CHF	EF: 32.85 ± 15.9%; EF: 35.53 ± 12.7%	E: 28 C: 16	E: 60 ± 8.7; 31 ± 7; 21 males C: 58.19 ± 12.8; 31 ± 6; 5 males	E: progressive, moderate intensity aerobic; C: education and flexibility and stretching exercises	Weeks 1-2: 30 min, 3/week, 60% max HR; Weeks 3-4: 45 min, 3/week, 60% max HR; Weeks 5-12: 45 min, 3/week, 70% max HR	ACS promoter methylation △	Butts et al. (2018)

CAD, coronary artery disease; CHF, chronic heart failure; CCT, control clinical trial; CT, clinical trial; CPET, cardiopulmonary exercise testing; EF, ejection fraction; HCS, healthy control subjects; OI, oxygen intervention; POAD, peripheral occlusive arterial disease; RCP, respiratory compensation point; RCT, randomized clinical trial.

### 4.1 DNA methylation in cardiovascular diseases

Denham and colleagues explored the epigenetic mechanisms by which sprint interval training, a form of exercise, improves cardiometabolic health (28). Genome-wide leukocyte DNA methylation changes were analyzed before and after a four-week period of sprint interval training in 12 young men using a 450 K BeadChip, and alterations in gene expression were verified. Results showed that participants had elevated cardiorespiratory fitness and athletic performance, decreased LDL cholesterol levels, and significant demethylation across the genome, particularly in CpG islands and gene promoter regions, suggesting that exercise promotes transcriptional activity of genes. In particular, altered methylation status of epidermal growth factor and associated miRNAs (e.g., miR-21 and miR-210) further affected the expression of genes related to cardiovascular function. This is the only interventional study to correlate exercise-induced epigenetic changes in human subjects with the cardiovascular system.

Zhang et al. explored the role of aerobic exercise in controlling hypertension and improving vascular function, specifically by modifying key ion channels in CVD through regulation of DNA methylation (29). Exercise reduced the expression and function of these ion channels by increasing the methylation of the genes for the *α*1c subunit of L-type Ca and the *β*1 subunit of large-conductance Ca-activated K, thereby contributing to the reduction of arterial blood pressure in hypertensive states. Exercise-induced alterations in DNA methylation mainly involve DNMTs and demethylases, and the activities of these enzymes are regulated by intermediate metabolites such as *α*-ketoglutarate and S-adenosylmethionine. These results imply that exercise regulates cardiovascular function by altering the epigenetic status of specific genes in vascular smooth muscle cells.

### 4.2 Histone modifications in cardiovascular diseases

Although studies of exercise-induced histone modifications in cardiovascular health are limited, there is evidence that strenuous exercise alters histone conformation in human skeletal muscle. The effects of exercise on histone modifications in human skeletal muscle, in particular the increased acetylation of histone H3K36, a modification associated with enhanced gene expression during transcriptional elongation (30). At the same time, exercise was found to lead to the export of specific histone deacetylases (e.g., HDAC4 and HDAC5) from the nucleus, thereby relieving their inhibitory effects on transcription. In addition, exercise activated signaling pathways such as AMPK and CaMKII, which are thought to promote the extra-nuclear export of HDAC4 and 5, thereby regulating histone acetylation status.

Future studies should further explore how exercise affects cardiovascular health through epigenetic mechanisms. In particular, studies should focus on how exercise can prevent or treat CVD by altering histone modifications in the cardiovascular system. With the advancement of epigenetic research techniques, future studies can also explore in detail how exercise meticulously regulates the expression of cardiovascular-related genes through higher precision methods. Exercise-induced changes in gene expression in cardiovascular cells could be investigated, for example, by single-cell sequencing, or the CRISPR/Cas9 system could be used to study the function of specific epigenetic marks in cardiovascular health.

### 4.3 Non-coding RNAs in cardiovascular diseases

Exercise can slow down the development of atherosclerotic plaques by repairing endothelial dysfunction and reducing local inflammatory death of endothelial cells (31). A new study finds that aerobic exercise can slow the development of atherosclerosis by modulating the long non-coding RNA (lncRNA) NEAT1 to reduce its expression (32). In NEAT1 knockout mice and human samples analyzed, exercise was shown to reduce the N6-methyladenosine (m6A) modification of NEAT1 and the activity of the related m6A-modifying enzyme METTL14. METTL14 promotes NEAT1 expression by recognizing its m6A site. In addition, NEAT1 drives endothelial cell pyroptosis by binding to the KLF4 transcription factor and activating the transcription of the key pyroptosis protein NLRP3. Therefore, by reducing the expression of NEAT1, exercise helps to inhibit endothelial cell death, thereby ameliorating atherosclerosis. This finding demonstrates the cardioprotective effect of exercise in downregulating NEAT1 expression through m6A modification and deepens our understanding of the complex mechanisms of exercise-mediated epigenetic alterations.

In an experiment on rats, researchers simulated a chronic heart failure (CHF) environment to observe the effects of eight weeks of aerobic exercise on heart health and its mechanisms (33). The results of the experiment showed that rats with CHF that underwent training showed significant cardiac function enhancement, particularly enhanced left ventricular function and reduced cardiac fibrosis, compared to untrained controls. Exercise simultaneously reduced reactive oxygen species and inflammatory factor levels and promoted cardiomyocyte autophagy. overexpression of the lncRNA MALAT1 may have counteracted the positive effects of exercise, suggesting that it may play a role by regulating miR-150–5p and the downstream PI3 K/Akt signaling pathway.

These results highlight the importance of lncRNAs in the regulation of CVD, particularly in exercise-mediated therapeutic strategies for heart disease, where noncoding RNAs may be key to cardioprotective mechanisms (33).

After inducing a myocardial infarction model by ligating the left anterior descending coronary artery, Farsangi et al. trained rats with moderate-intensity aerobic exercise for four weeks (34). The results showed that exercise significantly improved cardiac function and reduced myocardial fibrosis and apoptosis. Exercise acted by regulating the expression of specific lncRNAs, specifically decreasing MIAT and increasing H19 and GAS5 expression. Changes in these lncRNAs were strongly associated with improved cardiac contractile function and reduced myocardial fibrosis. These findings emphasize the potential mechanism of aerobic exercise in the management of cardiac health through the regulation of myocardial infarction-associated lncRNAs, revealing the positive impact of exercise on cardiac remodeling after myocardial infarction.

## 5 Future directions

Although many studies have noted epigenetic changes associated with cardiac health and disease, research on exercise-induced epigenetic responses remains limited. First, a large number of studies have focused on blood-based DNA methylation, and future research needs to focus on more relevant cell types and other types of epigenetic modifications. As technology advances and detection limits increase, we will be able to explore low abundance and other types of modifications in human DNA. Second, the relevance of epigenetic mechanisms observed in typical animal exercise models (e.g., moderate swimming or treadmill running) to other forms of human exercise (e.g., cycling or weight training) has not been well established. Third, existing systematic reviews examining the effects of various exercise intensities and durations on cardiac epigenetics have provided only preliminary insights (35), suggesting the need for more definitive studies. Finally, most of the current research suggests a correlation rather than a causal relationship between epigenetic modifications (e.g., DNA methylation, histone changes, and noncoding RNA) and the positive health effects of exercise. More rigorous investigations are needed to determine these associations.

## 6 Conclusion

Regular exercise undoubtedly provides significant health benefits, as it helps the body to adapt to physical loads. In recent years, it is increasingly recognized that an individual's response to exercise is influenced by genetic factors and epigenetic mechanisms. Exercise is an important stimulus that can be used to promote epigenetic changes that are beneficial to health, but the epigenetic landscape of exercise-induced cardioprotective effects is still in its infancy. Clarifying the epigenetic processes underlying the relationship between heart health and exercise will advance our understanding of exercise-based cardioprotection, contribute to the development of more specialized and innovative epigenetic therapies, and help to discover new biomarkers that will lead to the development of more effective therapies that will improve the quality of life of patients with CVD.
